# Supplementary material for: Enzyme-activating B-cell receptors boost antigen presentation to pathogenic T cells in gluten-sensitive autoimmunity
Source: Nat Commun. 2025 Mar 10;16:2387. doi: 10.1038/s41467-025-57564-5 (PMC11894174; doi:10.1038/s41467-025-57564-5)
Supplement: Supplementary file 1 — Supplementary Information [file 41467_2025_57564_MOESM1_ESM.pdf]

Supplementary information

Enzyme-activating B-cell receptors boost antigen presentation to pathogenic T cells in gluten-sensitive autoimmunity

Rasmus Iversen[1,2,#,*], Julie Elisabeth Heggelund[1,2,#], Saykat Das[1,2], Lene S. Høydahl[1,2,3], Ludvig M. Sollid[1,2,*]

[1] Norwegian Coeliac Disease Research Centre, Institute of Clinical Medicine, University of Oslo, Oslo, Norway
[2] Department of Immunology, Oslo University Hospital - Rikshospitalet, Oslo, Norway
[3] Present address: Nextera AS, Oslo, Norway

[#] Equally contributing authors

*Corresponding authors; rasmus.iversen@medisin.uio.no or l.m.sollid@medisin.uio.no

Supplementary Figures

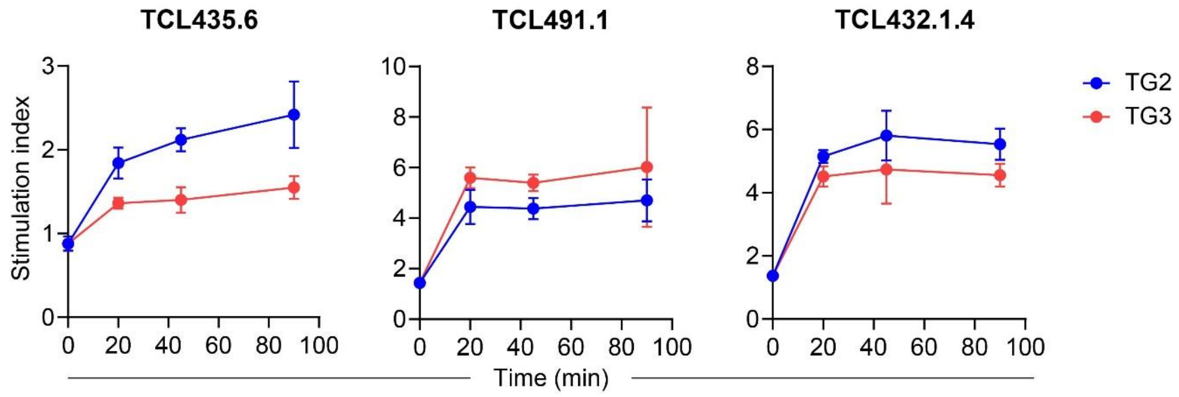

Supplementary Fig. 1: Generation of deamidated gluten epitopes by TG2 and TG3.
Chymotrypsin-digested gluten was treated with active TG2 or TG3 for various amounts of time before the enzymes were heat-inactivated. Timepoint zero represents untreated gluten without addition of TG2 or TG3. The gluten digest was used to stimulate three deamidation-dependent, gluten-reactive T-cell lines generated from gut biopsies of CeD patients. T-cell proliferation was assessed by ^{3}H-thymidine incorporation, and the stimulation index was calculated as the signal obtained with gluten divided by the signal obtained with medium only. Symbols represent means of culture triplicates and error bars indicate SD. Source data are provided as a Source Data file.

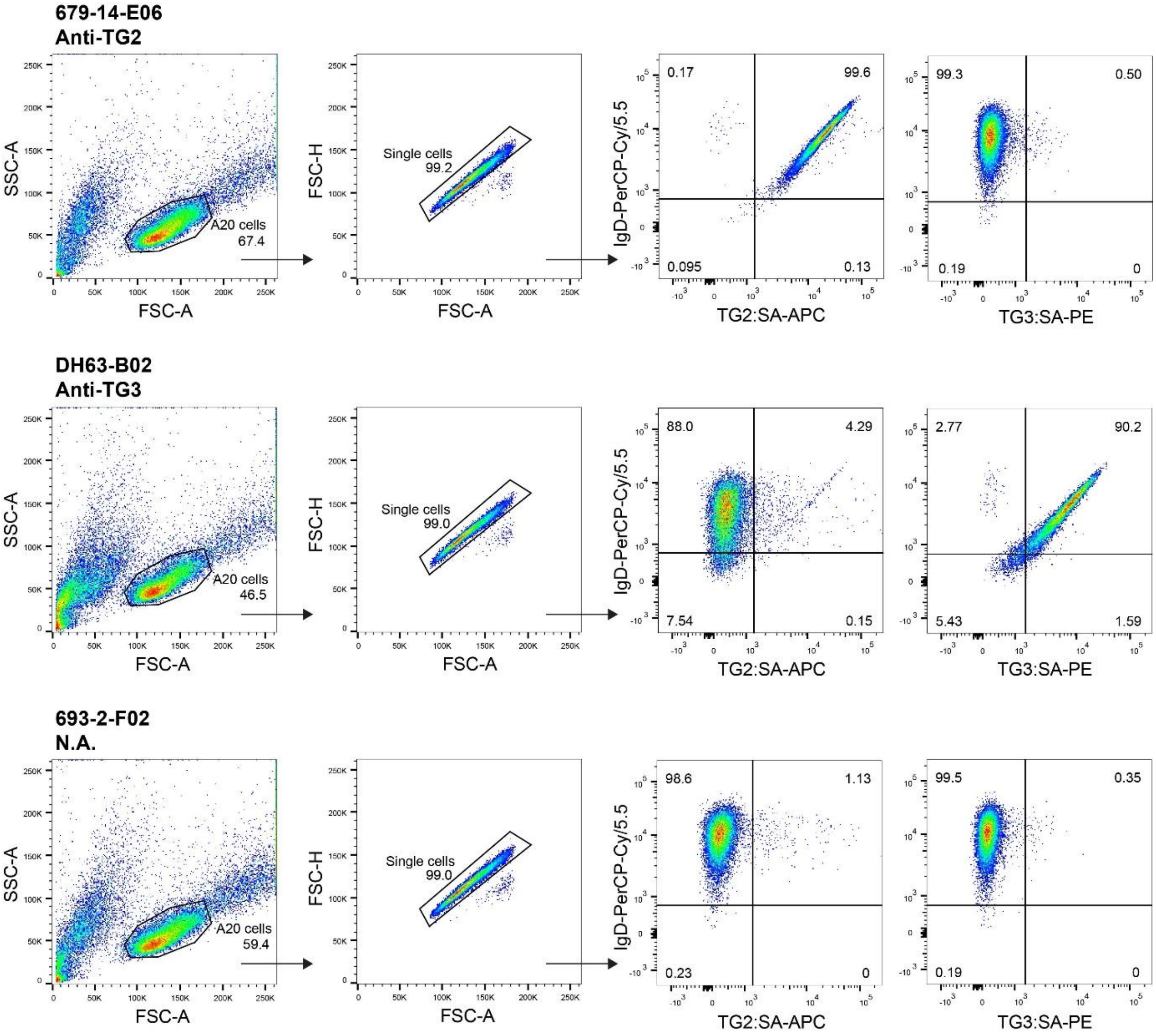

Supplementary Fig. 2: Gating strategy for BCR-transduced A20 B-cell lines.

Flow cytometry plots showing staining of A20 cells transduced with three different IgD BCR constructs. The cells were either specific for TG2 (679-14-E06), TG3 (DH63-B02) or had unknown specificity (693-2-F02). Each cell line was stained for surface IgD in combination with recombinant biotinylated antigens coupled to fluorescently labelled streptavidin (SA). N.A. not applicable.

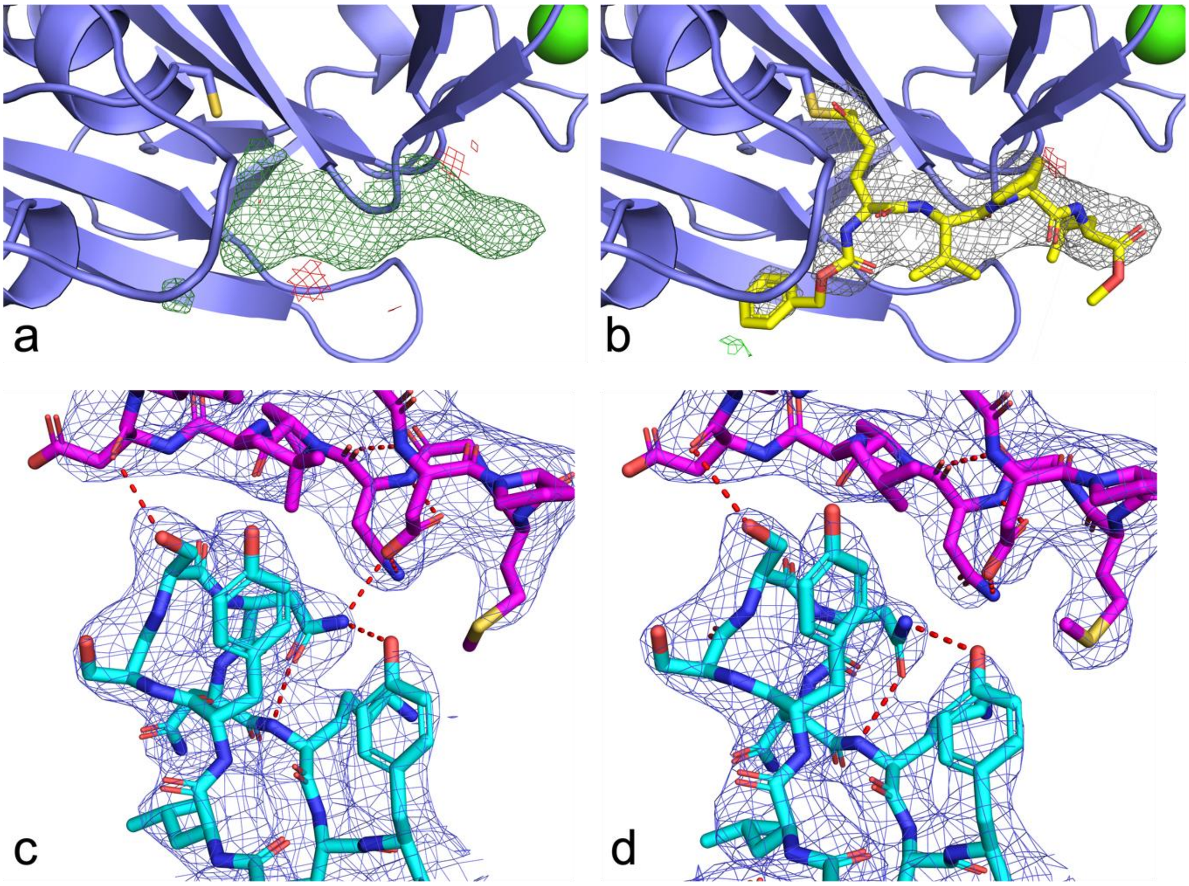

Supplementary Fig. 3: Electron density maps of TG3-bound Z-DON and Fab DH63-A02.
a, Difference density omit map of the active site of TG3 in the structure with bound Z-DON substrate-mimicking inhibitor (PDB ID: 8RMY). This Fo-Fc map was generated in Coot before the addition of Z-DON and is contoured in PyMol at 3.0 σ. **b**, 2Fo-Fc electron density map (grey, contoured at 1.0 σ) surrounding Z-DON in the final TG3 structure, overlayered with the difference density Fo-Fc map (green/red), contoured at 3.0 σ. **c,d,** 2Fo-Fc electron density maps (blue, contoured at 1.0 σ) of the interface between the CDR-L1 loop of Fab DH63-A02 (light blue sticks) and TG3 (magenta sticks) in the structure without inhibitor (**c,** PDB ID: 8RMX) or with Z-DON bound in the active site of TG3 (**d,** PDB ID: 8RMY). Notable hydrogen bonds are shown as red lines.

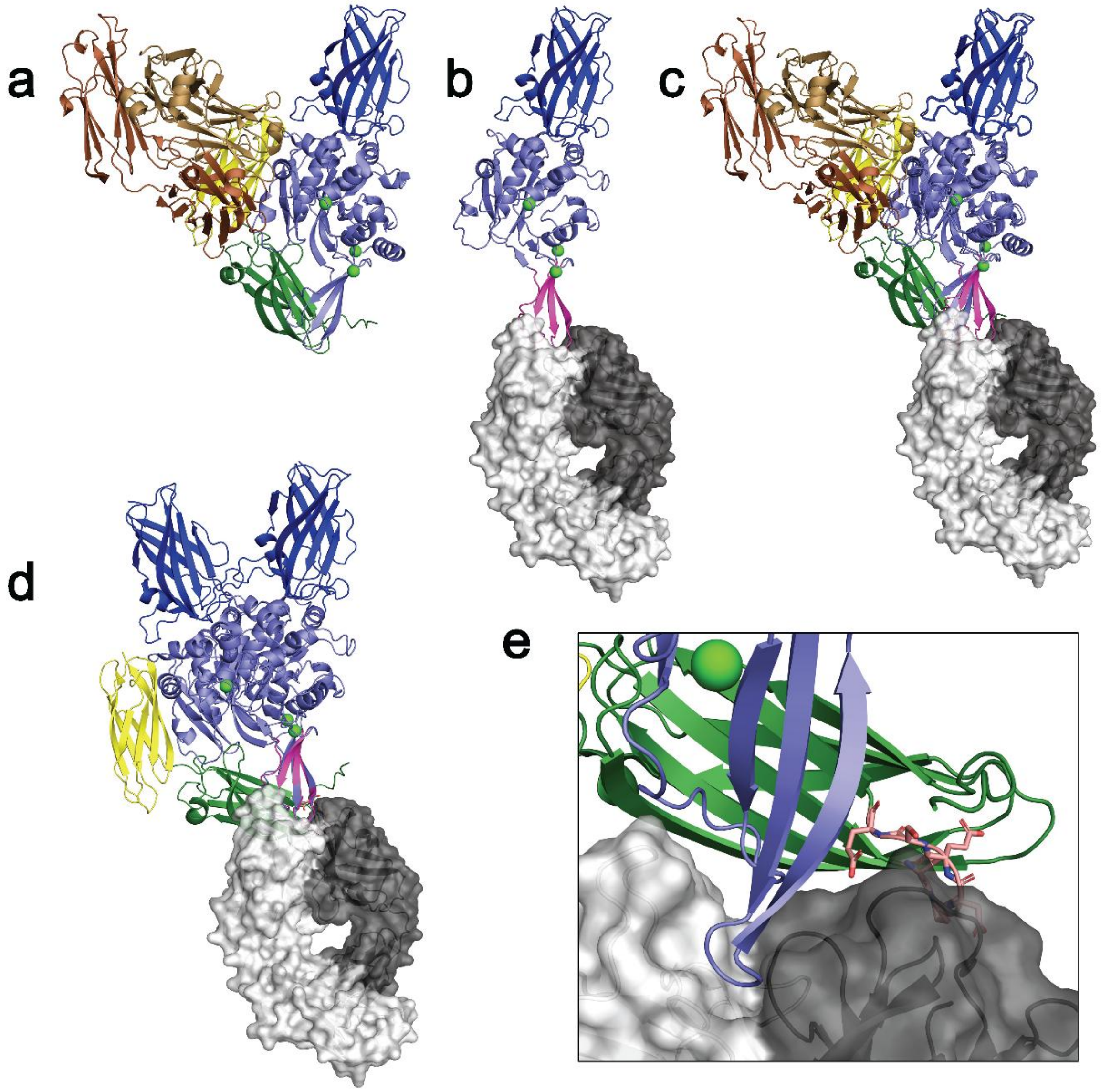

Supplementary Fig. 4: Predicted clash between the C1 domain of TG3 and Fab DH63-A02.
a, Crystal structure of TG3 bound to Fab DH63-B02 (previous work, PDB ID: 8OXW). TG3 is shown in dark blue (N-terminal domain), light blue (core domain), green (C1 domain) and yellow (C2 domain). The three calcium ions are shown as green spheres. Fab DH63-B02 is shown in brown cartoon representation. **b**, Crystal structure of TG3 bound to Fab DH63-A02 (PDB ID: 8RMX). The β-sheet that moves in the active conformation of TG3 is highlighted in magenta. DH63-A02 (grey/white) is shown in surface representation. **c**, Superimposition of the structures shown in **a** and **b**. **d**, Superimposition of only the TG3 catalytic core β-sheets of the structures in **a** and **b**. Fab DH63-B02 is omitted for clarity. **e**, Closeup of the predicted clash between TG3 domain C1 (green) and Fab DH63-A02 (white/grey). The clashing residues of TG3 are shown in salmon-colored stick representation.

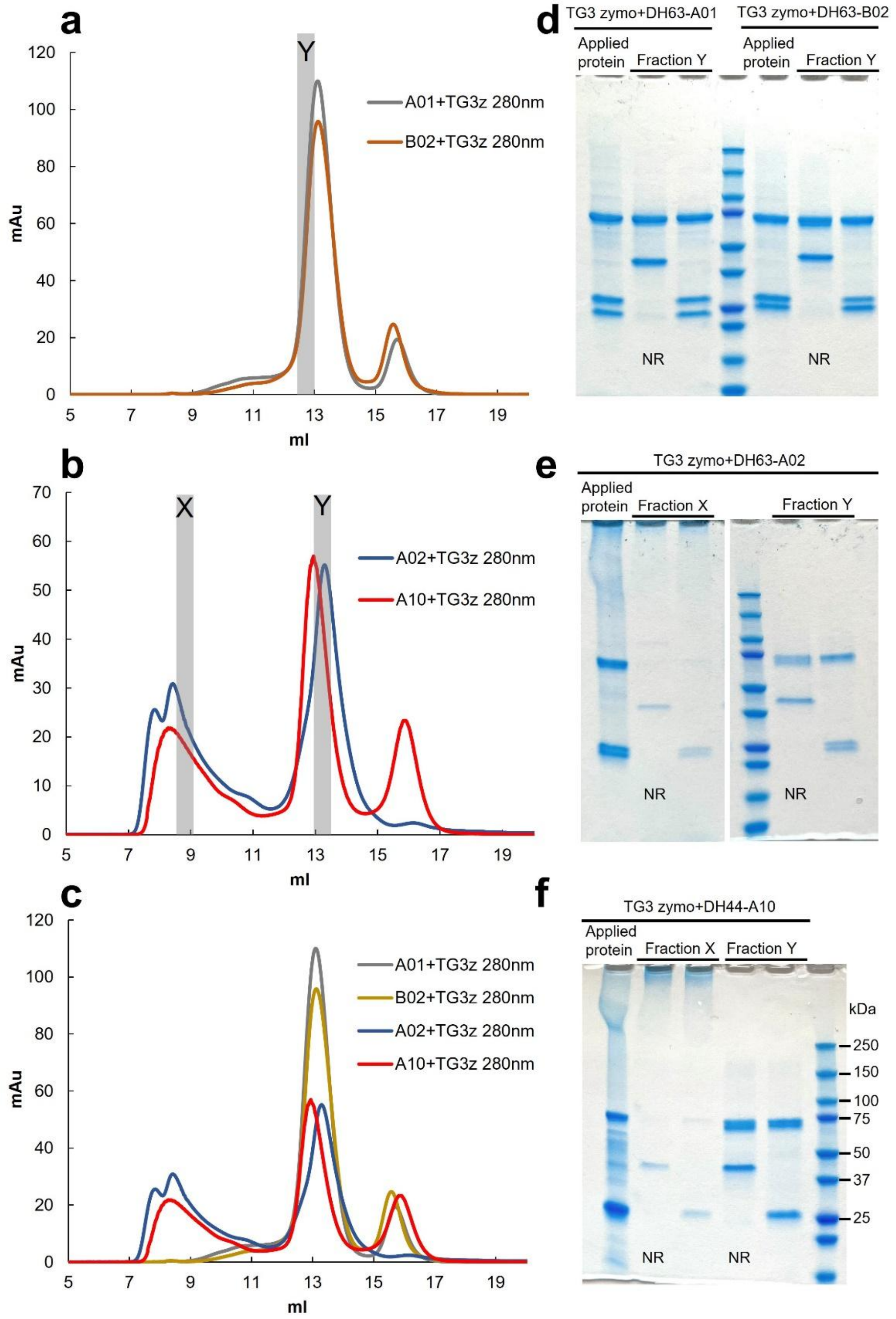

Supplementary Fig. 5: Effects of Fab binding on zymogen TG3.

a-**c**, Elution profiles (A280) of zymogen TG3 incubated with the indicated anti-TG3 Fabs obtained by size exclusion chromatography (SEC). Fabs targeting epitope 1 (DH63-A01) or epitope 2 (DH63-B02) are shown in **a**, and two Fabs targeting epitope 3 (DH63-A02 and DH44-A10) are shown in **b**. An overlay of all four TG3-Fab complexes is shown in **c**. **d**-**f**, SDS-PAGE analysis of the SEC fractions indicated in **a** and **b**. The samples were run under reducing or non-reducing (NR) conditions. Fraction Y contains non-covalent complexes of TG3 (80 kDa) and Fab (one band at 47 kDa under non-reducing conditions, and two bands at 23-25 kDa under reducing conditions). Fraction X contains high-MW covalent complexes generated through TG3-mediated transamidation. Source data are provided as a Source Data file.

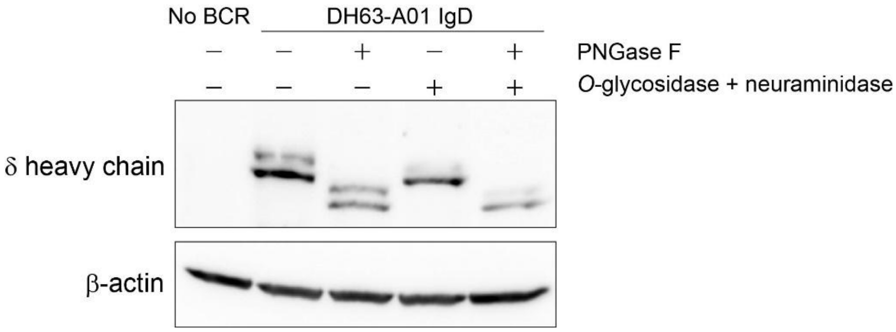

Supplementary Fig. 6: Glycoforms of IgD BCR.
Western blot showing detection of δ heavy chain or control protein (β-actin) in lysates of A20 cells with or without transduced anti-TG3 IgD BCR (DH63-A01). PNGase F was added to the lysate to remove *N*-linked glycans, whereas *O*-glycosidase and neuraminidase were used to cleave *O*-linked glycans. Only the upper band is affected by cleavage of *O*-linked glycans, while both bands shift size, when *N*-linked glycans are removed. One of two experiments is shown. Source data are provided as a Source Data file.

Supplementary Tables

Supplementary Table 1. Data collection and refinement statistics

**Complex**	TG3 + Fab DH63-A02	TG3 + Fab DH63-A02 + Z-DON
**PDB ID**	8RMX	8RMY
**Beam line**	Max IV BioMax	ESRF ID23-1
**Resolution (Å)**	132-2.8Å (2.86-2.80)	69.2-2.9Å (2.96-2.90)
**Space group**	I2	I2
**Unit cell**		
**a (Å)**	93.0	94.2
**b (Å)**	264.0	260.2
**c (Å)**	148.7	139.0
**β (°)**	106.3	95.1
**$CC_{1/2}$**	99.4 (37.9)	98.3 (18.5)
**Completeness (%)**	99.3 (87.7)	99.9 (99.9)
**No. of unique reflections**	84,425 (4064)	73,407 (4520)
**Multiplicity**	7.1 (7.1)	5.5 (5.3)
**$I / \sigma(I)$**	5.1 (0.5)	5.9 (0.6)
**R_{meas} (%)**	20.1 (330)	14.7 (238)
**R_{cryst}/R_{free} (%)**	20.1 / 24.8	19.0 / 23.2
**rmsd bond length (Å)**	0.012	0.010
**rmsd bond angles (°)**	1.152	1.012
**B-factors (Å^2)**		
**Backbone**	107.0	112.4
**side chains**	110.1	115.0
**Ligand**	-	116.9
**Ramachandran plot (%)**		
**Favored**	95.7	93.8
**Allowed**	4.0	5.9
**High-energy**	0.3	0.3

Supplementary Table 2. Interactions between Fab DH63-A02 and TG3

Fab DH63-A02	IMGT	Atom	TG3	Atom	Distance (Å) in 8RMX chain A	Distance (Å) in 8RMX chain D	Distance (Å) in 8RMY chain A	Distance (Å) in 8RMY chain D
Heavy chain CDR2								
Asp56	59	OD1/2	K408	NZ	3.06	3.32	2.81	2.70
Asp58	64	OD2	K408	NZ	2.88	3.16	3.20	3.24
Heavy chain FR3								
Arg60	66	NH2	T406	O	2.85	2.92	2.91	2.88
Arg60	66	NE	T406	O	2.89	2.89	2.81	2.86
Heavy chain CDR3								
Arg100	107	NH1/2	D403	OD2	2.57	2.16	2.60	2.68
Arg100	107	NH2	D403	OD1	2.84	2.97	2.92	3.15
Light chain CDR1								
Ser33	33	OG	D321	O	3.25	-	(3.41)	-
Asn34	34	ND2	D314	OD2	3.30	3.18	-	(3.91)
Light chain CDR3								
Tyr97	107	O	T405	OG1	2.68	2.57	2.39	2.03
Leu100	115	N	T405	O	3.34	3.22	3.14	3.01
Showing only H-bonds and electrostatic interactions with favorable angles and distances under 3.3 Å between donor and acceptor in at least one of the structures. “N” denotes the main chain nitrogen, and “O” denotes the main chain carbonyl. The rest are side chain atoms.								
