# Supplementary material for: Enzyme-activating B-cell receptors boost antigen presentation to pathogenic T cells in gluten-sensitive autoimmunity
Source: Nat Commun. 2025 Mar 10;16:2387. doi: 10.1038/s41467-025-57564-5 (PMC11894174; doi:10.1038/s41467-025-57564-5)
Supplement: Supplementary file 2 — Reporting Summary [file 41467_2025_57564_MOESM2_ESM.pdf]

nature portfolio

Corresponding author(s): Rasmus Iversen

Last updated by author(s): Feb 17, 2025

# Reporting Summary

Nature Portfolio wishes to improve the reproducibility of the work that we publish. This form provides structure for consistency and transparency in reporting. For further information on Nature Portfolio policies, see our Editorial Policies and the Editorial Policy Checklist.

## Statistics

For all statistical analyses, confirm that the following items are present in the figure legend, table legend, main text, or Methods section.

| n/a | Confirmed | |
|---|---|---|
| ☐ | ☒ | The exact sample size (*n*) for each experimental group/condition, given as a discrete number and unit of measurement |
| ☐ | ☒ | A statement on whether measurements were taken from distinct samples or whether the same sample was measured repeatedly |
| ☒ | ☐ | The statistical test(s) used AND whether they are one- or two-sided<br>*Only common tests should be described solely by name; describe more complex techniques in the Methods section.* |
| ☒ | ☐ | A description of all covariates tested |
| ☒ | ☐ | A description of any assumptions or corrections, such as tests of normality and adjustment for multiple comparisons |
| ☐ | ☒ | A full description of the statistical parameters including central tendency (e.g. means) or other basic estimates (e.g. regression coefficient) AND variation (e.g. standard deviation) or associated estimates of uncertainty (e.g. confidence intervals) |
| ☒ | ☐ | For null hypothesis testing, the test statistic (e.g. *F*, *t*, *r*) with confidence intervals, effect sizes, degrees of freedom and *P* value noted<br>*Give P values as exact values whenever suitable.* |
| ☒ | ☐ | For Bayesian analysis, information on the choice of priors and Markov chain Monte Carlo settings |
| ☒ | ☐ | For hierarchical and complex designs, identification of the appropriate level for tests and full reporting of outcomes |
| ☒ | ☐ | Estimates of effect sizes (e.g. Cohen's *d*, Pearson's *r*), indicating how they were calculated |

*Our web collection on statistics for biologists contains articles on many of the points above.*

## Software and code

Policy information about availability of computer code

| | |
|---|---|
| Data collection | The crystallographic data were collected at the European Synchrotron and Radiation Facility in Grenoble, France. Flow cytometry data was collected using BD FACSChorus or BD FACSDiva software. |
| Data analysis | The crystallographic data were analysed using the CCP4 software suite. The following programs were used:<br>-Aimless v0.7.13<br>-Phaser v2.8.3 /.2<br>-Refmac v5.8.0419<br>-Coot v0.9.8.92<br>-Dials v3.12.1<br>Details have been submitted to the Protein Data Bank (www.rcsb.org) and will be publicly available. Structural figures were prepared using PyMol v3.1.1. Biochemical data were analysed with GraphPad Prism v10, and flow cytometry data were analysed with FlowJo v10. |

For manuscripts utilizing custom algorithms or software that are central to the research but not yet described in published literature, software must be made available to editors and reviewers. We strongly encourage code deposition in a community repository (e.g. GitHub). See the Nature Portfolio guidelines for submitting code & software for further information.

# Data

Policy information about availability of data

All manuscripts must include a data availability statement. This statement should provide the following information, where applicable:

- Accession codes, unique identifiers, or web links for publicly available datasets
- A description of any restrictions on data availability
- For clinical datasets or third party data, please ensure that the statement adheres to our policy

Crystal structures of Fab DH63-A02 bound to TG3 with and without the substrate-mimicking inhibitor Z-DON attached to the active site have been deposited at the Protein Data Bank with PDB IDs 8RMY and 8RMX, respectively. The previously published structures 8OXX and 8OXW were used for comparison. Plasmids and cell lines generated in this study are available from the corresponding authors upon request. Source Data are provided with this paper.

# Research involving human participants, their data, or biological material

Policy information about studies with human participants or human data. See also policy information about sex, gender (identity/presentation), and sexual orientation and race, ethnicity and racism.

| | |
|---|---|
| Reporting on sex and gender | Not applicable, our study does not involve human participants |
| Reporting on race, ethnicity, or other socially relevant groupings | Not applicable, our study does not involve human participants |
| Population characteristics | Not applicable, our study does not involve human participants |
| Recruitment | Not applicable, our study does not involve human participants |
| Ethics oversight | Not applicable, our study does not involve human participants |

Note that full information on the approval of the study protocol must also be provided in the manuscript.

# Field-specific reporting

Please select the one below that is the best fit for your research. If you are not sure, read the appropriate sections before making your selection.

☒ Life sciences ☐ Behavioural & social sciences ☐ Ecological, evolutionary & environmental sciences

For a reference copy of the document with all sections, see nature.com/documents/nr-reporting-summary-flat.pdf

# Life sciences study design

All studies must disclose on these points even when the disclosure is negative.

| | |
|---|---|
| Sample size | The study describes the effect of anti-TG3 antibodies on TG3 catalytic activity. The sample size was limited by the number of monoclonal anti-TG3 antibodies available. These antibodies were reported in a previous study (Das et al., doi: https://doi.org/10.1101/2023.05.31.542741), which also identified three main epitope groups. Antibodies/BCRs representing each of these groups were further characterised in the current study. Notably, both clonotypes that we had available targeting epitope 3 were used for BCR construction to confirm that targeting of this epitope gives increased antigen uptake and presentation. A single epitope 3-specific Fab was used for co-crystallisation with TG3. |
| Data exclusions | No data were excluded from the analyses |
| Replication | All biochemical assays reported in the manuscript were repeated at least once. All replication attempts were succesful. |
| Randomization | Randomization is not relevant to our study, because we did not have individual study groups |
| Blinding | Blinding is not relevant to our study, because we did not have individual study groups |

# Reporting for specific materials, systems and methods

We require information from authors about some types of materials, experimental systems and methods used in many studies. Here, indicate whether each material, system or method listed is relevant to your study. If you are not sure if a list item applies to your research, read the appropriate section before selecting a response.

## Materials & experimental systems

| n/a | Involved in the study | |
| --- | --- | --- |
| ☐ | ☒ | Antibodies |
| ☐ | ☒ | Eukaryotic cell lines |
| ☒ | ☐ | Palaeontology and archaeology |
| ☒ | ☐ | Animals and other organisms |
| ☒ | ☐ | Clinical data |
| ☒ | ☐ | Dual use research of concern |
| ☒ | ☐ | Plants |

## Methods

| n/a | Involved in the study | |
| --- | --- | --- |
| ☒ | ☐ | ChIP-seq |
| ☐ | ☒ | Flow cytometry |
| ☒ | ☐ | MRI-based neuroimaging |

# Antibodies

| | |
| --- | --- |
| Antibodies used | Anti-TG3 antibodies cloned from duodenal plasma cells of DH patients (Das et al., doi: https://doi.org/10.1101/2023.05.31.542741)<br><br>Commercial antibodies:<br>Mouse anti-human IgD-PerCP/Cy5.5 (BioLegend, Cat# 348208; RRID:AB_10641706)<br>Goat F(ab')2 anti-human IgD (SouthernBiotech, Cat# 2032-01; RRID:AB_2795634)<br>Rabbit anti-human IgD (Dako, Cat# A0093; RRID:AB_3094764)<br>Rabbit anti-β-actin (Cell Signaling Technology, Cat# 8457; RRID:AB_10950489)<br>Goat anti-rabbit Ig-HRP (SouthernBiotech, Cat# 4010-05; RRID:AB_2632593) |
| Validation | The specificity of anti-TG3 antibodies has been validated in a previous study (Das et al., doi: https://doi.org/10.1101/2023.05.31.542741).<br><br>Information from vendors:<br><br>Mouse anti-human IgD-PerCP/Cy5.5: Human peripheral blood lymphocytes were stained with CD19 APC and IgD (clone IA6-2) PerCP/Cyanine5.5, or mouse IgG2a, κ PerCP/Cyanine5.5 isotype control.<br><br>Goat F(ab')2 anti-human IgD: Human peripheral blood lymphocytes were stained with Goat F(ab')2 anti-human IgD and mouse anti-human CD19-PE followed by swine anti-goat IgG, human/rat/mouse SP ads-FITC.<br><br>Rabbit anti-β-actin: Western blot analysis of extracts from various cell lines using β-actin (D6A8) rabbit mAb.<br><br>Goat anti-rabbit Ig-HRP: Lysates from mouse embryonic fibroblasts expressing no Bak, or WT human Bak were resolved by electrophoresis, transferred to nitrocellulose membrane, and probed with anti-bak followed by goat anti-rabbit Ig-HRP. |

# Eukaryotic cell lines

Policy information about cell lines and Sex and Gender in Research

| | |
| --- | --- |
| Cell line source(s) | Human T cell lines of CeD patients (all female, source: University of Oslo)<br>TCR-transduced BW58a–β– mouse hybridoma T cells (Qiao et al., doi: 10.4049/jimmunol.1101526 and Snir et al., doi: 10.1172/jci.insight.93961)<br>BCR-tranduced A20 mouse lymphoma B cells (Di Niro et al., doi: 10.1038/nm.2656 and Iversen et al., doi: 10.4049/jimmunol.1501363) |
| Authentication | The used cell lines were not authenticated |
| Mycoplasma contamination | The cell lines were tested for mycoplasma contamination and was confirmed to be negative. |
| Commonly misidentified lines (See ICLAC register) | No commonly misidentified lines were used. |

# Plants

| | |
| --- | --- |
| Seed stocks | N/A |
| Novel plant genotypes | N/A |
| Authentication | N/A |

## Flow Cytometry

### Plots

Confirm that:

☒ The axis labels state the marker and fluorochrome used (e.g. CD4-FITC).

☒ The axis scales are clearly visible. Include numbers along axes only for bottom left plot of group (a 'group' is an analysis of identical markers).

☒ All plots are contour plots with outliers or pseudocolor plots.

☒ A numerical value for number of cells or percentage (with statistics) is provided.

### Methodology

| | |
|---|---|
| Sample preparation | Flow cytometry was performed on A20 mouse lymphoma cells with or without transduced human BCRs. The cells were cultured at 37C, 5% CO2 in 10% (v/v) FBS/RPMI-1640 and harvested by centrifugation prior to staining. |
| Instrument | BD FACSMelody (cell sorting) or BD LSRFortessa (analysis) |
| Software | Data were collected using BD FACSChorus (FACSMelody) or BD FACSDiva (LSRFortessa) software and analysed with FlowJo v10. |
| Cell population abundance | BCR-transduced cells were sorted based on expression of surface IgD and binding of recombinant TG3. |
| Gating strategy | Cells were gated on FSC/SSC only |

☒ Tick this box to confirm that a figure exemplifying the gating strategy is provided in the Supplementary Information.
